# Supplementary material for: The Effect of Preoperative Administration of Glucocorticoids on the Postoperative Complication Rate in Liver Surgery: A Systematic Review and Meta-Analysis of Randomized Controlled Trials
Source: J Clin Med. 2024 Apr 3;13(7):2097. doi: 10.3390/jcm13072097 (PMC11012757; doi:10.3390/jcm13072097)
Supplement: Supplementary file 1 [file jcm-13-02097-s001.zip › jcm-2907353-supplementary/Supplement Table 2 - GRADE revised.pdf]

Author(s):

Question: Glucocorticoids compared to placebo or non-administration for liver surgery

Setting:

Bibliography:

Certainty assessment							№ of patients		Effect		Certainty	Importance
№ of studies	Study design	Risk of bias	Inconsistency	Indirectness	Imprecision	Other considerations	glucocorticoids	placebo or non-administration	Relative (95% CI)	Absolute (95% CI)		
**Overall postoperative complication rate (assessed with: events)**												
9	randomised trials	serious[a]	serious[b]	not serious	serious[c]	none	120/418 (28.7%)	149/419 (35.6%)	**OR 0.71** (0.38 to 1.31)	**74 fewer per 1,000** (from 182 fewer to 64 more)	⊕◯◯◯ Very low	CRITICAL
**Pleural effusion**												
5	randomised trials	serious[a]	serious[b]	not serious	not serious	none	23/328 (7.0%)	28/323 (8.7%)	**OR 0.81** (0.44 to 1.48)	**15 fewer per 1,000** (from 47 fewer to 36 more)	⊕⊕◯◯ Low	IMPORTANT
**wound infection**												
7	randomised trials	serious[a]	not serious	not serious	serious[c]	none	21/378 (5.6%)	32/367 (8.7%)	**OR 0.64** (0.45 to 0.92)	**30 fewer per 1,000** (from 46 fewer to 6 fewer)	⊕⊕◯◯ Low	IMPORTANT
**septic/infectious complications**												
4	randomised trials	serious[a]	serious[b]	serious[d]	serious[c]	none	22/300 (7.3%)	29/298 (9.7%)	**OR 0.73** (0.24 to 2.20)	**24 fewer per 1,000** (from 72 fewer to 94 more)	⊕◯◯◯ Very low	IMPORTANT
**bile leakage**												
7	randomised trials	serious[a]	not serious	not serious	serious[c]	none	35/378 (9.3%)	29/367 (7.9%)	**OR 1.12** (0.59 to 2.13)	**9 more per 1,000** (from 31 fewer to 75 more)	⊕⊕◯◯ Low	IMPORTANT
**liver failure**												
5	randomised trials	serious[a]	not serious	serious[c]	serious[c]	none	33/280 (11.8%)	33/271 (12.2%)	**OR 0.96** (0.49 to 1.88)	**4 fewer per 1,000** (from 58 fewer to 85 more)	⊕◯◯◯ Very low	IMPORTANT
**length of hospital stay**												
9	randomised trials	serious[a]	serious[b]	not serious	not serious	none	382	377	-	MD **0.12 fewer** (0.81 fewer to 0.58 more)	⊕⊕◯◯ Low	IMPORTANT

Certainty assessment							№ of patients		Effect		Certainty	Importance
№ of studies	Study design	Risk of bias	Inconsistency	Indirectness	Imprecision	Other considerations	glucocorticoids	placebo or non-administration	Relative (95% CI)	Absolute (95% CI)		
**total operative time**												
7	randomised trials	serious[a]	serious[b]	serious[e]	not serious	none	357	349	-	MD **2.82 lower** (19.48 lower to 13.83 higher)	⨁◯◯◯ Very low	IMPORTANT
**blood loss in mililiters**												
8	randomised trials	serious[a]	serious[a]	serious[f]	not serious	none	434	423	-	MD **3.41 higher** (37.84 lower to 44.67 higher)	⨁◯◯◯ Very low	IMPORTANT
**need for administration of blood products**												
5	randomised trials	serious[a]	serious[a]	not serious	not serious	none	41/288 (14.2%)	40/284 (14.1%)	**OR 1.04** (0.63 to 1.71)	**5 more per 1,000** (from 47 fewer to 78 more)	⨁⨁◯◯ Low	IMPORTANT

CI: confidence interval; **MD:** mean difference; **OR:** odds ratio

Explanations

a. RoB is considered serious due to the inclusion of high risk of bias articles.

b. Inconsistency between included studies is present.

c. Classification of outcomes should be performed with standardized measures.

d. This outcome should be classified using objective assessment measures, not clinical evaluation.

e. In the context of between-study heterogeneity, this outcome lack directness.

f. Given different types of surgeries, this pooled outcome lacks directness
