# Supplementary material for: The Effect of Preoperative Administration of Glucocorticoids on the Postoperative Complication Rate in Liver Surgery: A Systematic Review and Meta-Analysis of Randomized Controlled Trials
Source: J Clin Med. 2024 Apr 3;13(7):2097. doi: 10.3390/jcm13072097 (PMC11012757; doi:10.3390/jcm13072097)
Supplement: Supplementary file 1 [file jcm-13-02097-s001.zip › jcm-2907353-supplementary/Supplementary Table 1 - lab outcomes.pdf]

| Article | Study Type | Treatment Used | Treatment Dosage | Patients, control / intervention (#) | Total Bilirubin | ALT | AST | IL-6 | CRP | PTT |
|---|---|---|---|---|---|---|---|---|---|---|
| Onoe, S 2021 | RCT | Hydrocortisone | 500 mg immediately before hepatic pedicle clamping followed by 300 mg on POD 1, 200 mg on POD 2, and 100 mg on POD 3 | 46/48 | Significantly lower on POD2 (p=0.003) and POD3 (p=0.008) | Significantly lower on POD5 (p=0.019) and POD7 (p=0.045) | No significant difference on any POD | NR | Significantly lower on POD2 (p=<0.001), POD3 (p=<0.001), POD4 (p value not reported) | No significant difference on any POD |
| Steinthorsdottir, K. J. 2021 | RCT | Methylprednisolone | 10 mg/kg | 86/88 | Significantly lower on POD2 (p value not reported) and POD3 (p value not reported) | No significant difference on any POD | NR | NR | NR | Significantly lower on POD2 (p=0.024) |
| Bressan, A. K. 2022 | RCT | Methylprednisolone | 500 mg IV pre-operatively | 74/77 | Significantly lower on POD2 (p=0.03) | No significant difference on any POD | No significant difference on any POD | NR | NR | Significantly lower on POD2 (p=0.04) |
| Hasegawa, Y. 2019 | RCT | Methylprednisolone | 500 mg IV pre-operatively | 50/50 | Significantly lower on POD2 (p=<0.01) | Insufficient data for analysis | Insufficient data for analysis | Insufficient data for analysis | Significantly lower in intervention group* (p=<0.001) | Significantly lower in intervention group* (p=<0.35) |
| Donadon, M. 2016 | RCT | Methylprednisolone | 500 mg pre-operatively | 16/16 | Insufficient data for analysis | Insufficient data for analysis | NR | NR | NR | NR |

| | | | | | | | | | | |
|---|---|---|---|---|---|---|---|---|---|---|
| Hayashi, Y. 2011 | RCT | Hydrocortisone | 500 mg immediately before hepatic pedicle clamping followed by 300 mg on POD 1, 200 mg on POD 2, and 100 mg on POD 3 | 98/102 | Significantly lower on POD1 (p=<0.05), POD2 (p=<0.05), POD3 (p=<0.05), POD5 (p=0.01) | No significant difference on any POD | No significant difference on any POD | Significantly lower on POD0 (p=<0.05), POD1 (p=<0.05), POD3 (p=<0.05) | Significantly lower on POD2 (p=<0.05), POD3 (p=<0.05), POD5 (p=<0.05) | Significantly higher on POD1 (p=<0.05), POD2 (p=<0.05) |
| Yamashita, Y. 2001 | RCT | Methylprednisolone | 500 mg pre-operatively | 16/17 | Significantly lower on POD1 (p=0.02) | NR | NR | Significantly lower on POD1 (p=<0.05), POD3 (p=<0.05) | NR | NR |
| Muratore. A. 2003 | RCT | Methylprednisolone | 30 mg/kg | 28/25 | No significant difference on any POD | No significant difference on any POD | No significant difference on any POD | Significantly lower on POD1 (p=0.04) | NR | No significant difference on any POD |
| Aldrighetti, L. 2006 | RCT | Methylprednisolone | 500 mg pre-operatively | 36/37 | Significantly lower on POD1 (p=0.0001), POD2 (p=0.004), POD5 (p=0.0136) | Significantly lower on POD1 (p=0.02), POD2 (p=0.045) * | Significantly lower on POD1 (p=0.002), POD2 (p=0.027) * | Significantly lower on POD1, POD2 | NR | Significantly lower on POD1 (p=0.06), POD2 (p=0.026) |

| | | | | | | | | | | |
|---|---|---|---|---|---|---|---|---|---|---|
| Schmidt, S.C. 2007 | RCT | Methylprednisolone | 30 mg/kg | 10/10 | Significantly lower on POD6 (p=0.033) | No significant difference on any POD | NR | Significantly lower on POD1 (p=0.008), POD2 (p=0.002) | Significantly lower on POD1 (p=0.043), POD2 (p=0.002), POD3 (p=0.001), POD4 (p=0.014) | No significant difference on any POD |
| Turner, S. 2006 | RCT | Methylprednisolone | 10 mg/kg | 17/17 | NR | Significantly lower on POD2 (p=<0.0001) | NR | Insufficient data for analysis | NR | NR |
